# A Novel Strategy for Predicting 72-h Mortality After Admission in Patients With Polytrauma: A Study on the Development and Validation of a Web-Based Calculator

**DOI:** 10.3389/fmed.2022.799811

**Published:** 2022-04-14

**Authors:** Song Chen, Meiyun Liu, Di Feng, Xin Lv, Juan Wei

**Affiliations:** ^1^Department of Orthopaedic Trauma, East Hospital, School of Medicine, Tongji University, Shanghai, China; ^2^Department of Anesthesiology, Shanghai Pulmonary Hospital, School of Medicine, Tongji University, Shanghai, China

**Keywords:** injury severity score, Glasgow Coma Scale, base excess, lactate, polytrauma, mortality, nomogram

## Abstract

**Background:**

Early and accessible screening of patients with polytrauma at a high risk of hospital death is essential. The purpose of this research was to seek an accurate and convenient solution to predict deaths occurring within 72 h after admission of these patients.

**Methods:**

A secondary analysis was conducted on 3,075 patients with polytrauma from the Dryad database. We imputed missing values in eligible individuals with the k-nearest neighbor algorithm and then randomly stratified them into the training group (*n* = 2,461) and the validation group (*n* = 614) based on a proportion of 8:2. The restricted cubic spline, univariate, backward stepwise, and multivariate logistic regression methods were employed to determine the suitable predictors. Calibration and receiver operating characteristic (ROC) curves were applied to assess the calibration and discrimination of the obtained model. The decision curve analysis was then chosen as the measure to examine the clinical usage.

**Results:**

Age, the Glasgow Coma Scale score, the Injury Severity Score, base excess, and the initial lactate level were inferred as independent prognostic factors related to mortality. These factors were then integrated and applied to construct a model. The performance of calibration plots, ROC curves, and decision curve analysis indicated that the model had satisfactory predictive power for 72-h mortality after admission of patients with polytrauma. Moreover, we developed a nomogram for visualization and a web-based calculator for convenient application (https://songandwen.shinyapps.io/DynNomapp/).

**Conclusions:**

A convenient web-based calculator was constructed to robustly estimate the risk of death in patients with polytrauma within 72 h after admission, which may aid in further rationalization of clinical decision-making and accurate individual treatment.

## Introduction

Trauma is the leading cause of death and disability in the world. More than 5 million deaths annually are due to injuries from falls, traffic accidents, landslides, and explosions, among others. Patients with polytrauma are the main contributors to this figure, accounting for 65 to 72% of the cases (1, 2). These patients are often severely injured, which is associated with hemorrhagic or traumatic shock and immune dysfunction, requiring accurate assessment and rapid treatment. Moreover, early screening of patients at risk of in-hospital death is crucial for ensuring patient safety, allocating medical resources appropriately, and reducing healthcare costs (3).

At present, various trauma scoring systems and hematological tests are suitable for evaluating the overall prognosis of patients with multiple traumas, and the introduction of internal environmental indicators, such as initial blood lactate, base excess (BE), and pH, in particular, provides early predictive evaluations for clinical purposes (4–6). However, these independent assessment methods are tedious to calculate, have too many scoring criteria, and have limited predictive power in assessing patient prognosis in the initial phase of trauma. Development of a simple and easy-to-use predictive model that incorporates factors related to the high risk of early death in patients with polytrauma is desirable.

Of all the models available, the logistic regression approach can provide a personalized, evidence-based, highly precise risk estimation in classification tasks. In addition, the advent of nomograms and network calculators has made the models user-friendly for disease prognosis and prediction, which facilitates decision-making related to patient management (7–9). Inspired by these efforts, this study aimed to develop and validate a prediction model and wrap it into a web-based calculator that allows rapid and precise individualized prediction of the risk of death within 72 h in patients with multiple traumas, by incorporating a few easily accessible clinical predictors.

## Methods

### Data Source

The data sets yielded and analyzed are available from the Dryad Digital Repository, [https://datadryad.org/stash/dataset/doi.10.5061/dryad.bnzs7h45v]. The Dryad, an open resource database, provides a broad range of discoverable, freely reusable, and referable research data. Private information in the database has been anonymized. Data collection respects the principles outlined in the Declaration of Helsinki and has been approved by the local ethics committee.

### Study Design and Participants

A secondary retrospective analysis was performed based on the cohort study (10), which included multi-injury adult patients (>18 years old) treated at a Level I trauma center of the University Hospital Zurich from January 1, 1996 to January 1, 2013 and which excluded those with chronic diseases, oncological diseases, or genetic disorders that affect the musculoskeletal system. Time from injury to admission was defined as <24 h. Patients with multiple traumas were identified using an Injury Severity Score (ISS) of 16 or above, along with the criteria of the Berlin definition (11). Items selected from the data set for analysis are summarized in Table 1. The outcome was determined as patient's death within 72 h after admission. Related measurements of variables have been described carefully in the original article (10). Finally, among 3,668 patients recorded, 3,075 were enrolled, except for 579 (15.8%) with ISS values <16, 13 (0.4%) with no outcome data, and 1 (0.03%) with an incorrect body mass index (BMI) value marked as 0.

**Table 1:** Baseline characteristics of the training and validation sets.

**Characteristics**	**Training set** **(*N* = 2461)**	**Validation set** **(*N* = 614)**	*P* value^*^
Age, years, median (IQR)	43 (28, 61)	43 (28, 61)	0.917
Sex, n (%)			
Female	642 (26.1)	170 (27.7)	0.421
Male	1,819 (73.9)	444 (72.3)	
BMI, kg/m^∧^2,median (IQR)	24.7 (23.4, 26.1)	24.7 (23.4, 26.2)	0.687
ISS, median (IQR)	29 (22, 38)	27 (22, 36)	0.153
GCS, median (IQR)	6 (3, 14)	10 (3, 15)	0.225
pH, median (IQR)	7.34 (7.28, 7.38)	7.34 (7.29, 7.38)	0.400
BE, mmol/L, median (IQR)	−2.90 (−5.45, −1.10)	−2.70 (−5.30, −1.26)	0.632
Lactate, mmol/L, median (IQR)	2.30 (1.50, 3.50)	2.24 (1.50, 3.30)	0.195

* *P-values between groups were assessed by chi-square and Mann-Whitney tests*.

*BMI, body mass index; ISS, injury severity score; GCS, Glasgow Coma Scale; BE, base excess; IQR, interquartile range*.

### Missing Data

To maximize statistical power and minimize bias, k-nearest neighbor (KNN) (12) imputation with k equal to 10 was used to impute missing values in eligible patients. Then, the obtained imputation data were randomly stratified into two parts (i.e., training and validation cohorts) under a ratio of 8:2. We also carried out repeated analyses in the cohorts with complete data (i.e., data with all missing values removed) for comparison. Details on the statistical results are given in the Supplementary Material.

### Sample Size Calculation

The “*pmsampsize”* package of R, version 4.0.2 (http://www.r-project.org/), was utilized to calculate the minimum training sample size required. Eight candidate predictor parameters were chosen to construct a multivariable prediction model for the binary outcome. Moreover, based on previous evidence (10), outcome prevalence is anticipated to be 0.268 (26.8%), and a lower bound for the new model's R-squared value is 0.288. For the validation of sample size, a power calculation was carried out using PASS 15 (NCSS, LLC., Kaysville, UT, United States). The area under the receiver operating characteristic (ROC) curve (AUC; equivalent to the concordance statistic [C statistic]) was expected to be at least 0.8, and a two-tailed test with an alpha error of 0.05, beta error of 0.1, and power of 0.9 was conducted. As a result, the minimum sample size required for the training cohort is 302 patients with 81 events, while the validation cohort is 45 patients with 12 events. The eligible population is sufficient for model development and validation.

### Statistical Analysis

Continuous variables were expressed as medians with interquartile ranges (IQRs) and were compared by unpaired Mann-Whitney test. Categorical variables were compared by χ^2^ test. For each continuous variable at a significant level in the training cohort, we used a restricted cubic spline (RCS) with five knots at the 5, 35, 50, 65, and 95th percentiles to flexibly model its relationship with 72-h mortality after admission. Potential nonlinearity was tested using a likelihood ratio test comparing the model with only a linear term against the model with linear and cubic spline terms. Aiming to relax linear relationship assumptions, identified nonlinear continuous predictors were further categorized according to corresponding reference points determined by RCSs and horizontal lines with an odds ratio equal to 1. Then, linear continuous and acquired categorical predictors were examined with a univariate logistic regression approach for investigating the independent risk factors of mortality. All significant variables associated with death risks were candidates for stepwise multivariate analysis. To visualize the obtained model, a nomogram was generated according to multivariate logistic regression analysis outcomes and by applying the “*rms”* package. The predictive performance of the final model was measured by C statistic (13) and calibrated with 1,000 bootstrap samples for reducing overfitting bias. We also calculated the variance inflation factor (VIF) to examine the collinearity of each predictor in the prediction model and performed a formal sensitivity analysis, as described by Vander Weele and Ding (14), to capture the potential effect of unmeasured predictors on an obtained estimate.

For clinical utilization of the model, the total score for each patient was calculated from the nomogram. An ROC curve analysis was conducted to find optimal cutoff values that were determined by maximizing the Youden index (i.e., sensitivity + specificity−1). The accuracy of the optimal cutoff value was evaluated by the sensitivity, specificity, predictive values, and likelihood ratios. In addition, we established ROC curves for every predictor from the model. Pairwise comparisons of AUCs were tested with Delong's method. As a complement, decision curve analysis (DCA) was performed to quantify the clinical applicability of the model.

All the statistical analyses were completed with the R software. The remaining packages of R used were as follows: “*car*,” “*caret*,” “*splines*,” “*pROC*,” “*EValue*,” “*rmda*,” and “*ggplot2*”.

A two-tailed test was carried out to determine the level of statistical difference, and *p* < 0.05 was considered statistically significant except in pairwise comparison of AUCs. In this scenario, *p*-values were adjusted by Bonferroni correction and tested with a bound of 0.003.

## Results

### Baseline Characteristics

A total of 3,075 adult patients with polytrauma were entered in the design data set. To account for missing data, KNN imputation was performed for BMI in 1,501 (48.8%), the Glasgow Coma Scale (GCS) score in 43 (1.4%), pH in 832 (27.1%), base excess (BE) in 703 (22.9%), and lactate in 472 (15.3%). The median patient age was 43 (IQR 28–61) years. In total, 2,263 (73.6%) patients were men and 687 (22.3%) died within 72 h after admission. A similar population distribution was detected in the complete data, except for the mortality rate of 117 (11%). This discrepancy may be due to the removal of a large amount of missing information, resulting in a biased estimate (Supplementary Table S1).

Among the 3,075 patients, 2,461 and 614 were assigned to the training and validation groups, respectively. Baseline characteristic distributions were similar between the cohorts. Mortality was 550 (22.3%) and 137 (22.3%) patients in the 2 groups, respectively (Table 1).

Compared to survivors in the training cohort, those who died showed a higher rate of age (42 [IQR 27–58] vs. 51 [IQR 31–72], *P* < 0.001), BMI (24.6 [IQR 23.1–26.1] vs. 24.9 [IQR 24–26], *P* < 0.001), ISS (27 [IQR 21–34] vs. 34 [IQR 25–50], *P* < 0.001), and lactate (2.1 [IQR 1.4–3.04] vs. 3.3 [IQR 2.2–5.5], *P* < 0.001) and presented a lower value in the GCS score (12 [IQR 3–15] vs. 3 [IQR 3–3], *P* < 0.001), pH (7.35 [IQR 7.3–7.38] vs. 7.28 [IQR 7.19–7.35], *P* < 0.001), and BE (−2.55 [IQR −4.40–−0.9] vs. −5.39 [IQR −9.7–−2.4], *P* < 0.001). No statistical difference was detected in gender between the two cohorts (Table 2).

**Table 2:** Baseline characteristics of patients who died or survived in the training cohort.

**Characteristics**	**Alive** **(*N* = 1,911)**	**Dead** **(*N* = 550)**	*P* value^*^
Age, years, median (IQR)	42 (27, 58)	51 (31, 72)	<0.001
Sex, *n* (%)			
Female	484 (25.3)	158 (28.7)	0.110
Male	1,427 (74.7)	392 (71.3)	
BMI, kg/m^∧^2, median (IQR)	24.6 (23.1, 26.1)	24.9 (24.0, 26.0)	<0.001
ISS, median (IQR)	27 (21, 34)	34 (25, 50)	<0.001
GCS, median (IQR)	12 (3, 15)	3 (3, 3)	<0.001
pH, median (IQR)	7.35 (7.30, 7.38)	7.28 (7.19, 7.35)	<0.001
BE, mmol/L, median (IQR)	−2.55 (−4.40, −0.90)	−5.39 (−9.70, −2.40)	<0.001
Lactate, mmol/L, median (IQR)	2.10 (1.40, 3.04)	3.30 (2.20, 5.50)	<0.001

* *P-values between groups were assessed by chi-square and Mann-Whitney tests*.

*BMI, body mass index; ISS, injury severity score; GCS, Glasgow Coma Scale; BE, base excess; IQR, interquartile range*.

### Model Specifications and Predictors

**Figure 1:**
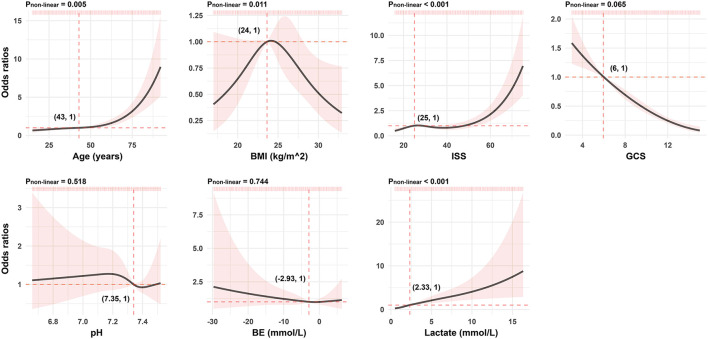
Association between continuous predictors and 72-h mortality in patients with polytrauma by the RCS analysis. For each curve, five knots at the 5th, 35th, 50th, 65th, and 95th percentiles were selected. Solid lines represent odds ratios, and shaded areas represent 95% CIs. The reference point is the first value closest or equal to the odds ratio at 1 (i.e., the intersection of the two red dashed lines). RCS, restricted cubic spline; BMI, body mass index; ISS, injury severity score; GCS, Glasgow Coma Scale; BE, base excess.

As presented in Figure 1, continuous variables like age, BMI, ISS, and lactate do not meet the linear relationship assumptions (All *P*_non−linear_ < 0.05). We converted these variables to categorical variables with reference points as cutoff values for the next univariable logistic analysis. Comparisons between those who survived and deceased patients were significant across all post-conversion variables (Table 3).

**Table 3:** Comparisons between patients who survived and deceased patients across all post-conversion variables in the training cohort.

**Variables**	**Alive** **(*N* = 1911)**	**Dead** **(*N* = 550)**	*P* value^*^
Age, years, *n* (%) <43 ≥43	978 (51.2) 933 (48.8)	215 (39.1) 335 (60.9)	<0.001
BMI, kg/m^∧^2,n (%) <24 ≥ 24	717 (37.5) 1,194 (62.5)	129 (23.5) 421 (76.5)	<0.001
ISS, *n* (%) <25 ≥ 25	679 (35.5) 1232 (64.5)	58 (10.5) 492 (89.5)	<0.001
Lactate, mmol/L, *n* (%) <2.33 ≥2.33	1,095 (57.3) 816 (42.7)	152 (27.6) 398 (72.4)	<0.001

* *P-values between groups were assessed by the chi-square test*.

*BMI, body mass index; ISS, injury severity score*.

The results of the univariate logistic analysis are shown in Table 4. Backward stepwise selection with AIC determined the following 6 variables that were most strongly associated with death risk: age, ISS, lactate, GCS, pH, and BE. In the multivariable analysis, age of at least 43 years old (OR 2.25; 95% confidence interval [CI] 1.78–2.83; *P* < 0.001), ISS of at least 25 (OR 2.96; 95% CI 2.15–4.07; *P* < 0.001), lactate of at least 2.33 (OR 2.16; 95% CI 1.67–2.81; *P* < 0.001), GCS score (OR.8; 95% CI 0.78–0.83; *P* < 0.001), and BE (OR 0.94; 95% CI 0.91–0.98; *P* = 0.001) were all independently related to mortality (Table 4). Similar findings are obtained in the complete data, as shown in Supplementary Table S3.

**Table 4:** A logistic regression analysis of the 72-h mortality for patients in the training cohort.

	**Univariable**		**Multivariable**	
**Variables**	**OR (95% CI)**	***P* value**	**aOR (95% CI)**	***P* value**
Factors selected by stepwise analysis				
Age, years <43 ≥43	1 [Reference] 1.63 (1.35, 1.98)	NA <0.001	1 [Reference] 2.25 (1.78, 2.83)	NA <0.001
ISS <25 ≥25	1 [Reference] 4.66 (3.52, 6.28)	NA <0.001	1 [Reference] 2.96 (2.15, 4.07)	NA <0.001
Lactate, mmol/L			
<2.33 ≥2.33	1 [Reference] 3.51 (2.86, 4.33)	NA <0.001	1 [Reference] 2.16 (1.67, 2.81)	NA <0.001
GCS	0.78 (0.76, 0.81)	<0.001	0.80 (0.78, 0.83)	<0.001
pH	0.003 (0.001, 0.007)	<0.001	0.32 (0.08, 1.23)	0.097
BE, mmol/L	0.86 (0.84, 0.88)	<0.001	0.94 (0.91, 0.98)	0.001
Factors not selected by stepwise analysis				
BMI, kg/m^∧^2 <24 ≥24	1 [Reference] 1.96 (1.58, 2.44)	NA <0.001	NA	NA

*BMI, body mass index; ISS, injury severity score; GCS, Glasgow Coma Scale; BE, base excess; OR, odds ratio; aOR, adjusted odds ratio; CI, confidence interval; NA, not applicable*.

### Model Development and Validation

**Figure 2:**
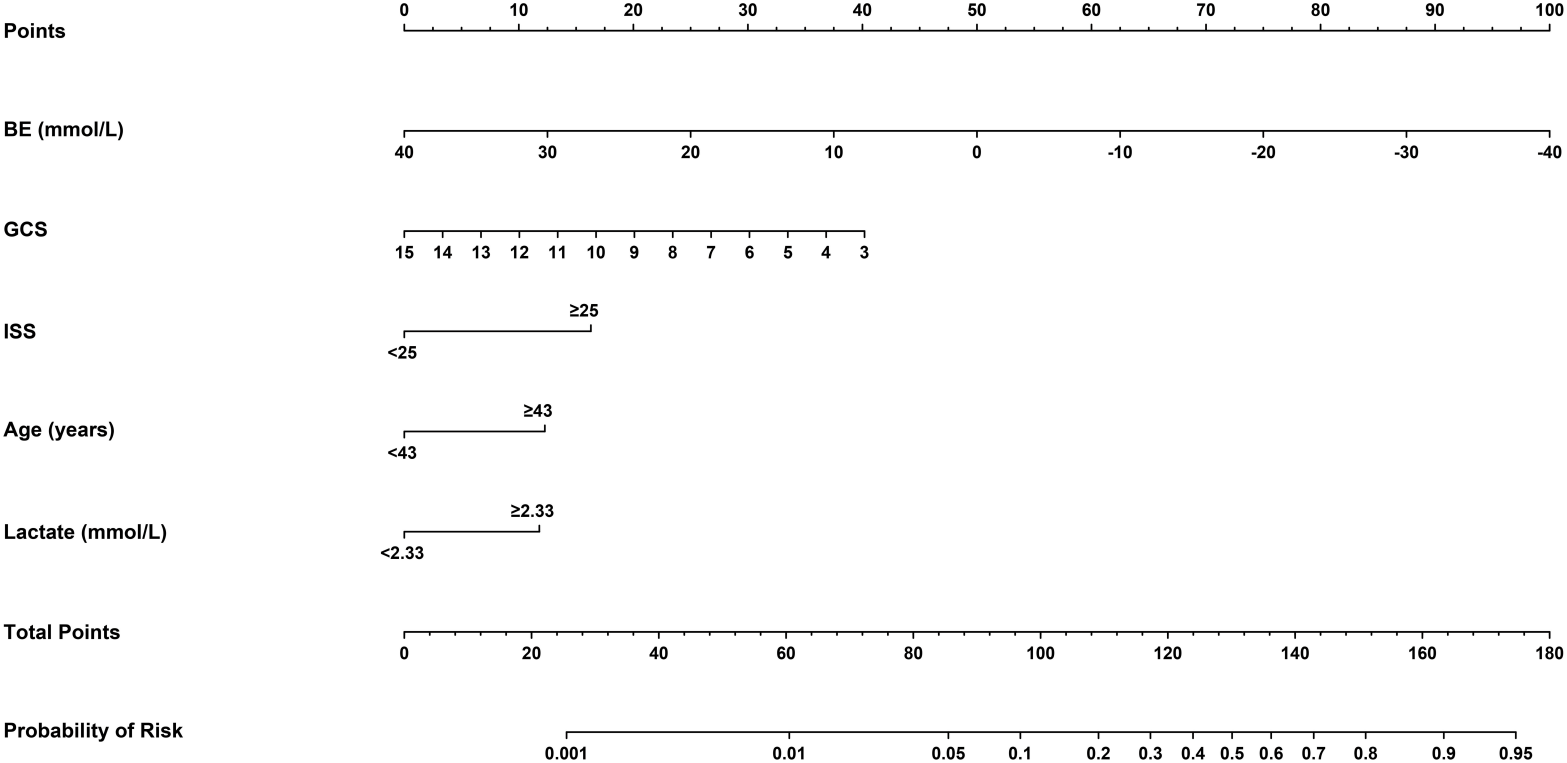
Nomogram for estimating the risk of 72-h mortality after admission in patients with polytrauma. Using a nomogram, we first determined the location of the axis corresponding to each variable and drew a vertical line to the “Points” axis to get a score, then, summed scores from all variables, and drew another vertical line from the “Total Points” axis to the “Probability of Risk” axis for the predicted probability. BE, base excess; GCS, Glasgow Coma Scale; ISS, injury severity score.

The identified independently associated risk factors were then applied to construct the final model and form a nomogram for estimating 72-h mortality risk after admission (Figure 2). For predictors from the model, VIFs were 2.76 or less, indicating the absence of collinearity. Moreover, the E-value, a standard way to quantify the potential effect of unmeasured predictors on the obtained estimate, for each predictor is calculated and presented in Supplementary Figure S1. The lowest one is 1.21; that is to say, our estimates were robust to unmeasured confounders, except in the case of a strong unmeasured confounder that was substantially associated with death risk. In order to simplify the clinical application of the model, we also designed a web-based calculator (https://songandwen.shinyapps.io/DynNomapp/) to predict death risks for patients with polytrauma (Supplementary Figure S2).

**Figure 3:**
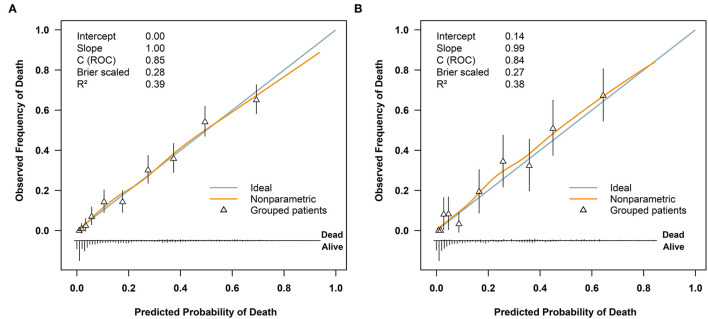
Receiver operating characteristic curves for validating the discrimination of our model and included variables: **(A)** in the training cohort (*n* = 2461) and **(B)** in the validation cohort (*n* = 614). ISS, injury severity score; GCS, Glasgow Coma Scale; BE, base excess.

Regarding model performance testing, we first completed internal validation with the bootstrap method. The model had good discrimination in assessing mortality, with an unadjusted C statistic of 0.85 (95% CI 0.83–0.86) and a bootstrap-corrected C statistic of 0.85. Besides, the calibration plots indicated a good agreement between risk estimates and actual deaths (Figure 3A), whereas in the validation data set, the model presented a C statistic of 0.84 (95% CI 0.81–0.88) for predicting mortality. There was also a satisfactory calibration curve for risk estimation (Figure 3B).

**Figure 4:**
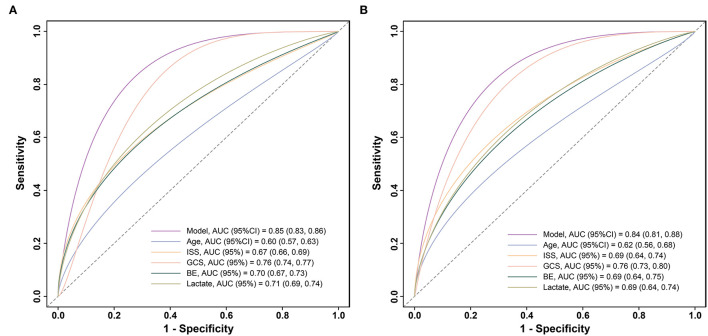
Calibration plots of the model for predicting 72-h mortality in patients with polytrauma **(A)** in the training cohort (*n* = 2461) and **(B)** in the validation cohort (*n* = 614). ROC, receiver operator characteristic.

To further compare the predictive value of the model, age, ISS, GCS, BE, and lactate for the 72-h mortality in patients with polytrauma, ROC curves were plotted (Figure 4). In the training cohort, relative AUCs were 0.85 (95% CI:0.83–0.86),0.6 (95% CI:0.57–0.63),0.67 (95% CI:0.66–0.69),0.76 (95% CI:0.74–0.77),0.7 (95% CI:0.67–0.73), and 0.71 (95% CI:0.69–0.74). There were no statistical differences among ISS, GCS, BE, and lactate (all *P* > 0.003), while differences between our model and any of the others were statistically significant (all *P* < 0.001) as well as age (*P* < 0.001). As for validation, the AUCs showed modest changes and were 0.84 (95% CI:0.81–0.88),0.62 (95% CI:0.56–0.68),0.69 (95% CI:0.64–0.74),0.76 (95% CI:0.73–0.8),0.69 (95% CI:0.64–0.75), and 0.69 (95% CI:0.64–0.74). Differences between the model and any of the others remained statistically significant (all *P* < 0.001), but no significance was observed in the remaining comparisons (all *P* > 0.003). The above evidence suggested that our model had superior predictive performance over any of the single predictors mentioned.

### Clinical Usage of the Model

We assumed that a patient with a nomogram score above a defined threshold was at high death risk, but that with the defined threshold was at low death risk. Then, a total score was calculated for each patient and an optimal cutoff value of 111 was determined. On this basis, sensitivity, specificity, positive predictive value (PPV), and negative predictive value (NPV) were 81.5, 74, 47.4, and 93.3% in the training cohort and 82.5, 73.8, 47.5, and 93.6% in the validation cohort, respectively (Table 5).

**Table 5:** Performance metrics of the nomogram for estimating the risk of 72-h mortality after admission.

	**Value (95% CI)**	
**Performance metrics**	**Training set**	**Validation set**
Cutoff score^*^	111	111
Sensitivity, %	81.5 (77.9, 84.6)	82.5 (75.1, 88.4)
Specificity, %	74.0 (72.0, 75.9)	73.8 (69.6, 77.7)
Positive predictive value, %	47.4 (44.9, 53.0)	47.5 (42.4, 59.5)
Negative predictive value, %	93.3 (91.8, 93.9)	93.6 (90.4, 94.8)
Positive likelihood ratio	3.13 (2.88, 3.41)	3.15 (2.66, 3.73)
Negative likelihood ratio	0.25 (0.21, 0.30)	0.23 (0.16, 0.34)

* *Optimal cutoff scores were determined by maximizing the Youden index (i.e., sensitivity + specificity−1). CI, confidence interval*.

**Figure 5:**
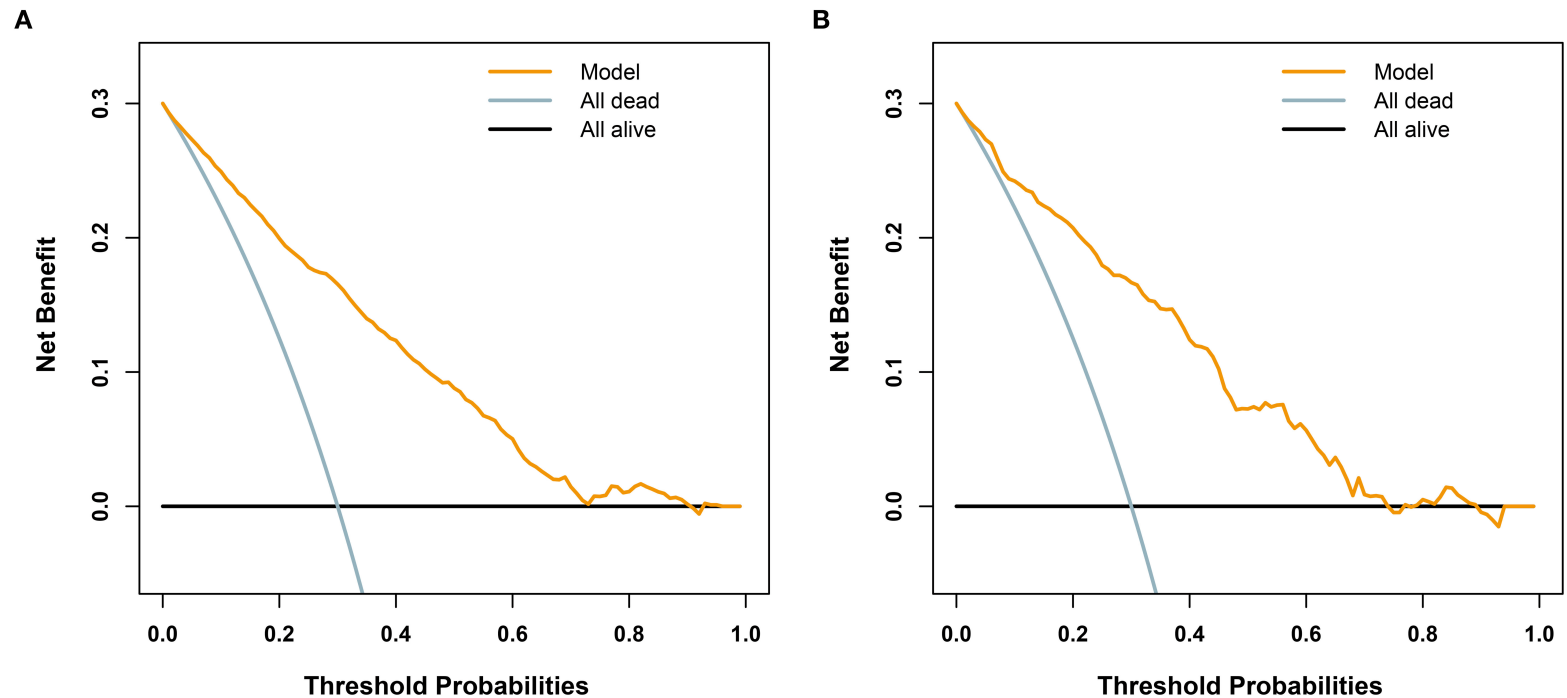
Decision analysis curves for the developed model for predicting 72-h mortality in patients with polytrauma. The black line represents a scheme to make all patients alive. The light blue line represents an improper protocol, leading to all patient mortality that occurred during the course of the study. The yellow line represents net benefits of the clinical application of the model. **(A)** In the training cohort (*n* = 2461), when the threshold is between 0.01 and 0.9, the model is applicable, since the net benefit is >0. **(B)** In the validation cohort (n = 614), the valid range is between 0.01 and 0.74.

Furthermore, we utilized DCAs to evaluate the net benefit of the model for decision-making. As illustrated in Figure 5, in the training cohort, the model is applicable when the threshold is between 0.01 and 0.9, as net benefits are >0, while the validation cohort has a valid range of between 0.01 and 0.74.

## Discussion

It is vital to evaluate the clinical condition of patients with multiple traumas in an early stage. From the views of most authors, estimating early mortality plays an equally important role in predicting subsequent complications (15–18). In this study, age, ISS, GCS, BE, and lactate were identified as independent risk factors for early death in patients with polytrauma based on the information extracted from an online database. The proposed model, which incorporated the above 5 readily accessible variables, performed impressively, being backed by C statistic values of 0.85 and 0.84 in the training and validation cohorts, respectively, and calibration curves indicated an excellent agreement between predictions and actual observations. Furthermore, our results revealed that age, ISS, GCS, BE, and lactate were less accurate in predicting mortality than our new model. To adapt the model to clinical practice, we summarized sensitivity, specificity, NPV, and PPV for assessing mortality risk by considering 111 as the cutoff value (Table 5). Patients with scores of 111 or more (1,185 of 3,075, [38.5%]) are selected into the high-risk subgroup. The corresponding net benefit values are 0.19 in the training cohort and 0.2 in the validation cohort. The abovementioned evidence support that our model might serve as an efficient and reliable tool for estimating the risk of 72-h death after admission and might aid in clinical decision-making.

The Glasglow Coma Scale and the ISS are routine initial assessment scoring systems for patients with polytrauma. The former, an essential measurement of neurological function and severity of the head injury, has advantages such as being simple, practical, time-efficient, and cost-effective (19). Several authors have determined that low GCS was associated with poor outcome (20–22), which is consistent with our evidence from the RCS of GCS in Figure 1. The ISS is another major predictor of trauma mortality and focuses on anatomical scoring. Contrary to the GCS scores, the probability of patient survival decreases with increasing ISS scores (23). Watts et al. (24) reported that ISS was positively associated with in-hospital mortality in elderly patients with trauma. It is not unique. In our findings, compared to patients with ISS <25, the risk of death was approximately three-fold greater in patients with larger values. However, the above scoring systems fail to assess both physiological disorders and anatomical damages in patients with polytrauma. In addition, there is always confusion on how to scientifically synthesize the results of multiple scoring systems to guide next clinical implementations. Fortunately, none of these are issues for the model-based network calculator, and all it takes is to correctly categorize ISS values and determine the corresponding GCS values.

Age is also a predictor for the risk of mortality related to multiple injuries but not a part of the above two scoring systems (25). In a meta-analysis of older adults with trauma, Hashmi et al. (26) found that the risk of death increased with age and was two times higher in patients aged 74 than in those aged 62. We agree with the efforts of Hashmi et al. In addition, we noticed that the risk kept increasing faster when the patient was over 50 years old (Figure 1). In comparison with those younger than 43, the older ones were exposed to an additional 2.25-fold risk of death (OR 2.25; 95% CI 1.78–2.83). This reminds us that more attention should be paid to both middle-aged and older patients with multiple injuries. The subtle gap between our results and those of Hashmi et al. (26) is possibly due to various observed populations and different statistical methods chosen for analysis. In any case, it is certainly a sensible move to include age in our model to improve the accuracy of the estimates.

Admission BE is a recognized trauma marker that can evaluate injury severity and forecast post-traumatic outcomes (27). Several studies have shown that an initial negative BE predicts the mortality risk of patients with trauma, meaning that the poorer the BE, the higher the in-hospital mortality (28–30). Across such research, we could observe a trend toward higher mean BE in survival than in death, which is also reflected in our study (−2.55 [IQR −4.40–−0.0] vs. −5.39 [IQR −9.7–−2.4], *P* < 0.001). Lichtveld et al. (28) concluded that BE was an independent predictor of mortality in patients with trauma, with an OR of 0.92 (95% CI:0.89–0.95), indicating an 8% increase in the risk of death for each unit reduction in BE. Our findings were in close agreement with those published by Lichtveld et al. The OR is 0.94 (95% CI:0.91–0.98), suggesting that every 1 mmol/L decrease in BE was associated with a 6% increase in the risk of death. These slight discrepancies may be explained by the different enrollment populations of the two studies. Furthermore, the effect of BE on 72-h mortality was also assessed by the ROC analysis. However, the measured AUCs were still significantly lower than the fit of the model we developed.

Lactate is a usual clinical biomarker for diagnosing shock and monitoring resuscitation. It is valuable not only for patients with sepsis shock (31) but also for patients with trauma. In a study including 1,829 patients with blunt trauma, Gale et al. (32) confirmed that the initial lactate was a dependable prognosticator of patients at a higher risk of in-hospital mortality. In another observational cohort study with 1,075 patients with trauma, Raux et al. (33) observed that admission lactate was superior in predicting early deaths, severe traumatic lesions, and massive hemorrhage. According to our research, patients with admission lactate over the bound of 2.33 mmol/L were at 2.16-fold risk of 72-h death than others (OR 2.16; 95% CI 1.67–2.81). The AUC of ROC used to estimate the predictive value of lactate for 72-h mortality was 0.71 in the training group and 0.69 in the validation group, which is close to 0.716 reported by Sammour (34) and lower than the performance of our model.

Moreover, we opted for a logistic regression approach to construct the prediction model, which may be limited by its linearity assumption. Although great care has been taken to build RCS models for exploring this assumption, the residual predictor-response variable complex relationship may still have been overlooked. These challenges may be easily solved by machine learning algorithms, which do not require assumptions of strict data structure and have the ability to learn complex functional forms with nonparametric methods. Furthermore, ensemble modeling methods, i.e., combining two or more machine learning algorithms, can be applied for the improvement of prediction accuracy (35). We are considering the application of this promising technology as an alternative in future research.

### Limitations

Our study has several limitations. First, in the final prediction model, missing data were present for the BMI, GCS, pH, BE, and lactate variables. The data were considered to be missing at random, and KNN imputation was conducted to minimize selection bias. We repeated the analysis on the complete data and obtained similar results. Second, this is a secondary analysis with fixed data, and we may have overlooked some important predictors. Therefore, we quantified the unmeasured factors to assess the robustness of our model. Third, all the analyses were conducted on the basis of data from one institution; there is a need to validate the results from other centers. Further prospective studies are also required to affirm the dependability of our model. Hence, the web calculator was developed to make these requirements easy to implement. In addition, although the model reached good predictive accuracy with a cutoff of 111, the false positive and false negative rates were 26 and 18.5% in the training cohort and 26.2 and 17.5% in the validation cohort, respectively. For 72-h mortality prediction, the performance of our model still needs to be improved to make meaningful clinical decisions.

## Conclusions

A clinical prediction model was constructed and wrapped into a web-based calculator to estimate mortality risk easily and robustly in patients with polytrauma within 72 h of hospital admission, which may contribute to further rationalization of clinical decision-making and accurate individual treatment. Under another aspect, the calculator may identify patients at a high risk of death and thus avoid corresponding adverse events.

## Data Availability Statement

The data utilized and analyzed in the current study is available in the Dryad database. The website is https://doi.org/10.5061/dryad.bnzs7h45v.

## Ethics Statement

The studies involving human participants were reviewed and approved by the Ethics Committees of the East Hospital of Tongji University, Shanghai, China. Written informed consent for participation was not required for this study in accordance with the national legislation and the institutional requirements.

## Author Contributions

SC, ML, DF, and JW designed the study. SC, ML, and JW performed data analysis and prepared the manuscript. JW and XL contributed to improving the article and funding for the project. All authors reviewed the manuscript, read, and approved the submitted manuscript.

## Funding

This study was supported by grants from the Clinical Research Plan of SHDC (No. SHDC2020CR3044B) and the Clinical Research Special Project of Shanghai Municipal Health Commission (No. 202040004).

## Conflict of Interest

The authors declare that the research was conducted in the absence of any commercial or financial relationships that could be construed as a potential conflict of interest.

## Publisher's Note

All claims expressed in this article are solely those of the authors and do not necessarily represent those of their affiliated organizations, or those of the publisher, the editors and the reviewers. Any product that may be evaluated in this article, or claim that may be made by its manufacturer, is not guaranteed or endorsed by the publisher.

## Abbreviations

KNN: k-nearest neighbor
BMI: body mass index
ISS: Injury Severity Score
GCS: Glasgow Coma Scale
BE: base excess
IQR: interquartile range
OR: odds ratio
aOR: adjusted odds ratio
CI: confidence interval
ROC: receiver operator characteristic
AUC: area under the ROC curve
RCS: restricted cubic spline
VIF: variance inflation factor
DCA: decision curve analysis
PPV: positive predictive value
NPV: negative predictive value.
