# Supplementary material for: A Novel Strategy for Predicting 72-h Mortality After Admission in Patients With Polytrauma: A Study on the Development and Validation of a Web-Based Calculator
Source: Front Med (Lausanne). 2022 Apr 14;9:799811. doi: 10.3389/fmed.2022.799811 (PMC9046941; doi:10.3389/fmed.2022.799811)
Supplement: Supplementary file 1 [file Data_Sheet_1.docx]

**Supplementary Materials**

**Table of contents.**

| **Item** | **Description** | **Page** |
| --- | --- | --- |
| Table S1 | Details of complete and imputation datasets. | 2 |
| Table S2 | Baseline characteristics of patients who died or survived in the complete data. | 3 |
| Table S3 | Logistic regression analysis of the 72-hour mortality for patients in the complete data. | 4 |
| Figure S1 | Bias plots for variables in nomogram model. | 5 |
| Figure S2 | Snapshots of a dynamic web-based calculator for the 72-hour mortality in polytrauma patients. | 5 |

Table S1. Details of complete and imputation datasets.

|  | Missing  N (%) | Complete  (N = 1062) | Imputation  (N = 3075) |
| --- | --- | --- | --- |
| Age, years, median (IQR) | NA | 41 (27, 56) | 43 (28, 61) |
| Sex, n (%) | NA |  |  |
| female |  | 264 (24.9) | 812 (26.4) |
| male |  | 798 (75.1) | 2263 (73.6) |
| BMI, kg/m^2, median (IQR) | 1501 (48.8) | 24.5 (22.3, 26.5) | 24.7(23.4, 26.1) |
| ISS, median (IQR) | NA | 29 (22, 38) | 27 (22, 38) |
| GCS, median (IQR) | 43 (1.4) | 12 (3, 15) | 7 (3, 14) |
| pH, median (IQR) | 832 (27.1) | 7.34 (7.27;7.39) | 7.34 (7.28;7.38) |
| BE, mmol/L, median (IQR) | 703 (22.9) | -2.90 (-5.40, -0.70) | -2.87 (-5.40, -1.14) |
| Lactate, mmol/L, median (IQR) | 472 (15.3) | 2.20 (1.40, 3.30) | 2.30 (1.50, 3.46) |
| Outcome, n (%) | NA |  |  |
| Alive |  | 945 (89.0) | 2388 (77.7) |
| Death |  | 117 (11.0) | 687 (22.3) |

BMI, body mass index; ISS, injury severity score; GCS, glasgow coma scale; BE, base excess; IQR, interquartile range; OR, odds ratio; NA, not applicable.

Table S2. Baseline characteristics of patients who died or survived in the complete data.

| Characteristics | Alive  (N = 945) | Dead  (N = 117) | *P* Value* |
| --- | --- | --- | --- |
| Age, years, median (IQR) | 41 (27, 55) | 47 (30, 65) | 0.009 |
| Sex, n (%) |  |  |  |
| female | 237 (25.1) | 27 (23.1) | 0.636 |
| male | 708 (74.9) | 90 (76.9) |  |
| BMI, kg/m^2, median (IQR) | 24.5 (22.2, 26.4) | 24.7 (22.8, 27.3) | 0.340 |
| ISS, median (IQR) | 29 (22, 36) | 33 (25, 43) | 0.001 |
| GCS, median (IQR) | 13 (3, 15) | 3 (3, 3) | <0.001 |
| pH, median (IQR) | 7.34 (7.28, 7.39) | 7.30 (7.22, 7.36) | <0.001 |
| BE, mmol/L, median (IQR) | -2.70 (-5.00, -0.60) | -4.90 (-8.40, -2.30) | <0.001 |
| Lactate, mmol/L, median (IQR) | 2.10 (1.39, 3.17) | 3.46 (2.23, 5.70) | <0.001 |

* *P* values between groups were assessed by the chi-square and Mann-Whitney test.

BMI, body mass index; ISS, injury severity score; GCS, glasgow coma scale; BE, base excess; IQR, interquartile range.

Table S3. Logistic regression analysis of the 72-hour mortality for patients in the complete data.

|  | Univariable |  |  | Multivariable |  |
| --- | --- | --- | --- | --- | --- |
| Variables | OR (95% CI) | *P* Value |  | aOR (95% CI) | *P* Value |
| Age, years  < 43  ≥ 43 | 1 [Reference]  1.50 (1.02, 2.23) | NA  0.039 |  | 1 [Reference]  2.07 (1.35, 3.18) | NA  < 0.001 |
| ISS  < 25  ≥ 25 | 1 [Reference]  3.04 (1.81, 5.44) | NA  < 0.001 |  | 1 [Reference]  2.15 (1.20, 3.86) | NA  < 0.001 |
| Lactate, mmol/L  < 2.33  ≥ 2.33 | 1 [Reference]  3.29 (2.17, 5.06) | NA  < 0.001 |  | 1 [Reference]  2.39 (1.46, 3.94) | NA  < 0.001 |
| GCS | 0.80 (0.76, 0.84) | < 0.001 |  | 0.81 (0.77, 0.85) | < 0.001 |
| pH | 0.038 (0.009, 0.156) | < 0.001 |  | 1.05 (0.10, 11.25) | 0.970 |
| BE, mmol/L | 0.90 (0.87, 0.93) | < 0.001 |  | 0.95 (0.89, 1.02) | 0.141 |

BMI, body mass index; ISS, injury severity score; GCS, glasgow coma scale; BE, base excess; OR, odds ratio; aOR, adjusted odds ratio; CI, confidence interval; NA, not applicable.

**Figure legends**

**Figure S1.** Bias plots for variables in nomogram model. RR_EU_ is the risk ratio for the relationship between measured variables (i.e., exposure variables) and unmeasured confounders, and RR_UD_ for the relationship of unmeasured confounders to the outcome. The E-value is a continuous measure of how robust the observed association is to potential uncontrolled confounders. The lowest possible E-value is 1 (i.e., no unmeasured confounding is needed to explain away the observed association). The higher the E-value the stronger the confounder-outcome association would have to be to explain away the effect. ISS, injury severity score; GCS, Glasgow coma scale; BE, base excess.

**Figure S2.** Snapshots of a dynamic web-based calculator for the 72-hour mortality in polytrauma patients. (A) Homepage (https://songandwen.shinyapps.io/DynNomapp/). Values of continuous variables can be chosen by using the slider, and categories corresponding to the categorical variables can be selected by pulling down the drop-down checkbox. Then click on the "Predict" button and predicted probabilities with confidence intervals will be presented in the graph. (B) We can check out results through switching to the “Numeric Summary” column. BE, base excess; GCS, Glasgow coma scale; ISS, injury severity score; Ge, greater and equal to; Lt, less than.
