# Supplementary figures and images for: A Novel Strategy for Predicting 72-h Mortality After Admission in Patients With Polytrauma: A Study on the Development and Validation of a Web-Based Calculator
Source: Front Med (Lausanne). 2022 Apr 14;9:799811. doi: 10.3389/fmed.2022.799811 (PMC9046941; doi:10.3389/fmed.2022.799811)

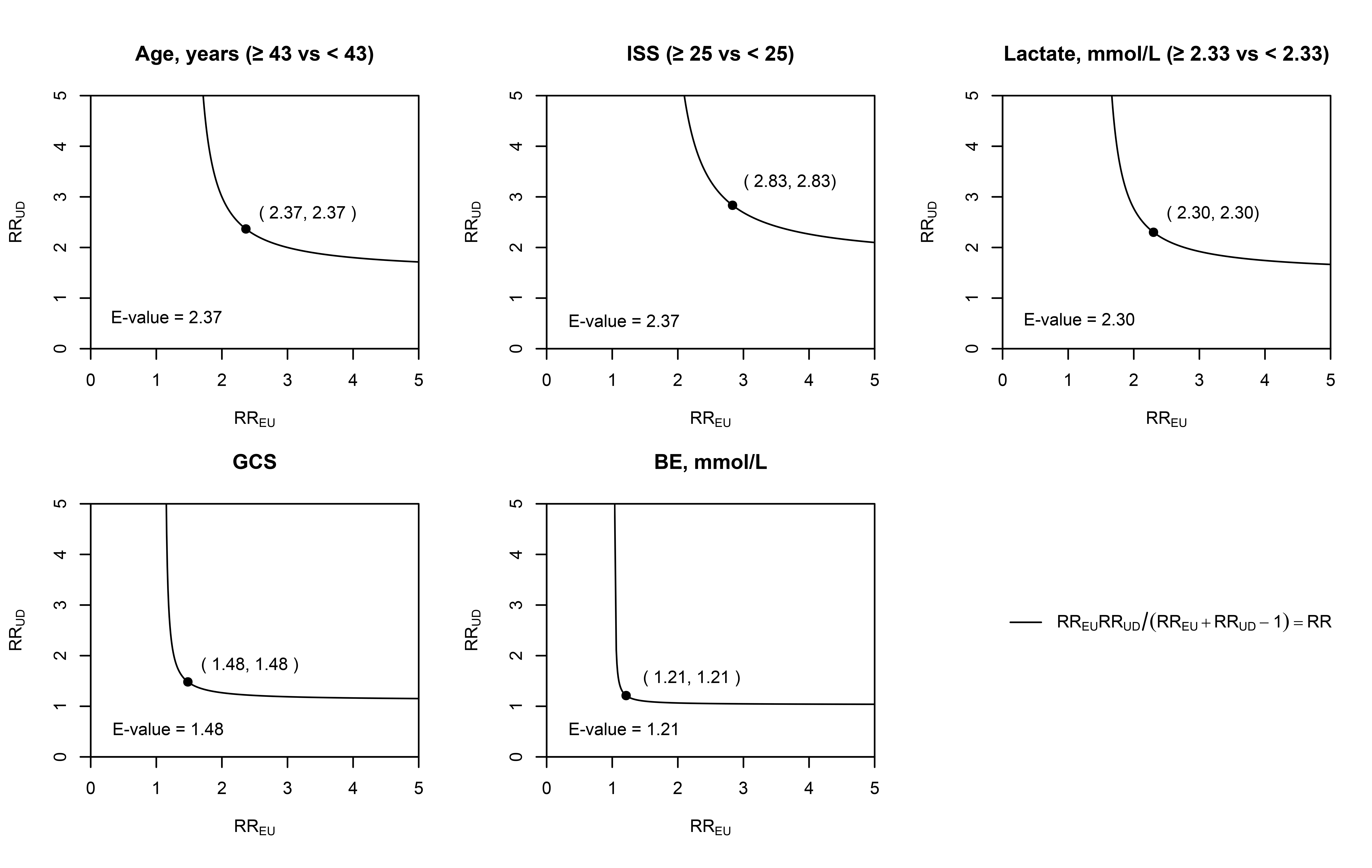

Supplement: Supplementary file 2 [file Image_1.tif]

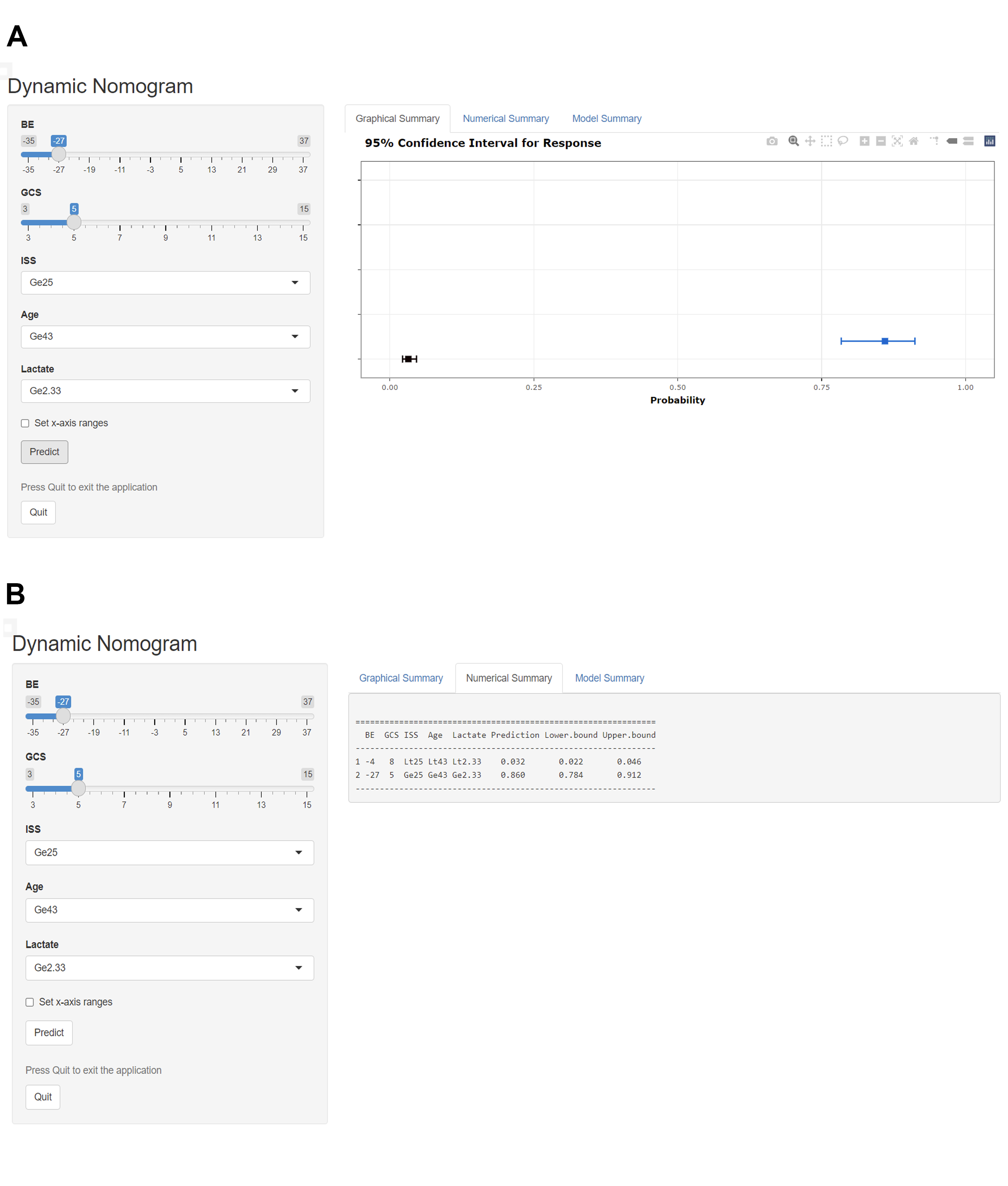

Supplement: Supplementary file 3 [file Image_2.tif]
